# Large-scale spatiotemporal spike patterning consistent with wave propagation in motor cortex

**DOI:** 10.1038/ncomms8169

**Published:** 2015-05-21

**Authors:** Kazutaka Takahashi, Sanggyun Kim, Todd P. Coleman, Kevin A. Brown, Aaron J. Suminski, Matthew D. Best, Nicholas G. Hatsopoulos

**Affiliations:** 1 Department of Organismal Biology and Anatomy, University of Chicago, 1025 E 57th Street Culver Room 206, Chicago, Illinois 60637, USA; 2 Committee on Computational Neuroscience, University of Chicago, 1025 E 57th Street Culver Room 206, Chicago, Illinois 60637, USA; 3 Department of Bioengineering, University of California, San Diego, 9500 Gilman Drive MC 0412, San Diego, La Jolla, California, 92093, USA; 4 Center for Neuroscience, New York University, 4 Washington Place, New York City, New York 10003, USA; 5 Department Electrical Engineering and Computer Science, Milwaukee School of Engineering, 1025 N Broadway, Milwaukee, Wisconsin 53202, USA

## Abstract

Aggregate signals in cortex are known to be spatiotemporally organized as propagating waves across the cortical surface, but it remains unclear whether the same is true for spiking activity in individual neurons. Furthermore, the functional interactions between cortical neurons are well documented but their spatial arrangement on the cortical surface has been largely ignored. Here we use a functional network analysis to demonstrate that a subset of motor cortical neurons in non-human primates spatially coordinate their spiking activity in a manner that closely matches wave propagation measured in the beta oscillatory band of the local field potential. We also demonstrate that sequential spiking of pairs of neuron contains task-relevant information that peaks when the neurons are spatially oriented along the wave axis. We hypothesize that the spatial anisotropy of spike patterning may reflect the underlying organization of motor cortex and may be a general property shared by other cortical areas.

## Abstract (web summary)

Aggregate signals in cortex are spatiotemporally organized as propagating waves across the cortical surface. Here the authors demonstrate that neurons in primary motor cortex of monkeys spatially coordinate their spiking activity in a manner that closely matches wave propagation.

## Graphical abstract

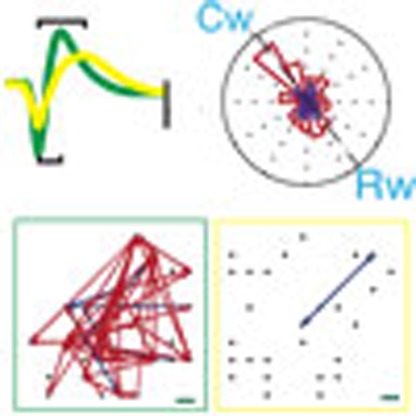

Propagating waves of neural activity are ubiquitous and have been documented at different spatial resolutions in a number of different neocortical areas including visual^1^,^2^,^3^,^4^,^5^,^6^, somatosensory^5^,^7^,^8^,^9^, auditory^10^,^11^ and motor cortices^12^,^13^,^14^ as measured via multielectrode local field potential (LFP) recordings, voltage-sensitive dyes (VSDs) and multiunit activities. Oscillatory LFPs and electroencephalograms in the beta frequency range (15–40 Hz) are ubiquitous in the motor cortex of mammals including monkeys^15^,^16^,^17^,^18^ and humans^19^,^20^. In particular, we have previously demonstrated that across the precentral gyrus of the upper-limb area of primary motor cortex (MI), these oscillations are not perfectly synchronized but rather exhibit phase gradients that indicate planar propagating waves along what we define as a beta wave axis, a rostro–caudal axis in monkeys^13^ and a medio–lateral axis in humans^14^ at a range of propagating speeds that were consistent across subjects.

However, as both LFPs and VSD measure aggregate potentials from groups of neurons near the recording site, it has never been shown whether action potentials from individual neurons demonstrate spatiotemporal patterning consistent with wave propagation. This is important because it is still debated as to what aggregate signals such as LFPs and VSD signify physiologically, whereas single-unit action potentials are understood to mediate interneuronal communication. Moreover, the functional significance of this wave propagation for motor control is unclear (but see recent computational studies^21^,^22^). Here we first show that MI neurons can be classified, based on the spike waveform widths, into two groups of neurons exhibiting distinct spectral properties. We then estimate effective connectivity of networks of spiking neurons based on this classification using a Granger causality analysis applied to point processes, and demonstrate that a class of simultaneously recorded, single-motor cortical neurons with narrow spike waveforms in non-human primates spatially coordinates their spiking activity in a manner that closely matches the orientation of prominent beta wave propagation. We also demonstrate that sequential spiking activity of that class of neuron pairs contains task-relevant, target-direction information whose magnitude varies according to the spatial orientation of the constituent neurons in a manner consistent with the beta wave axis.

## Results

### Beta waves in the motor cortex

**Figure 1:**
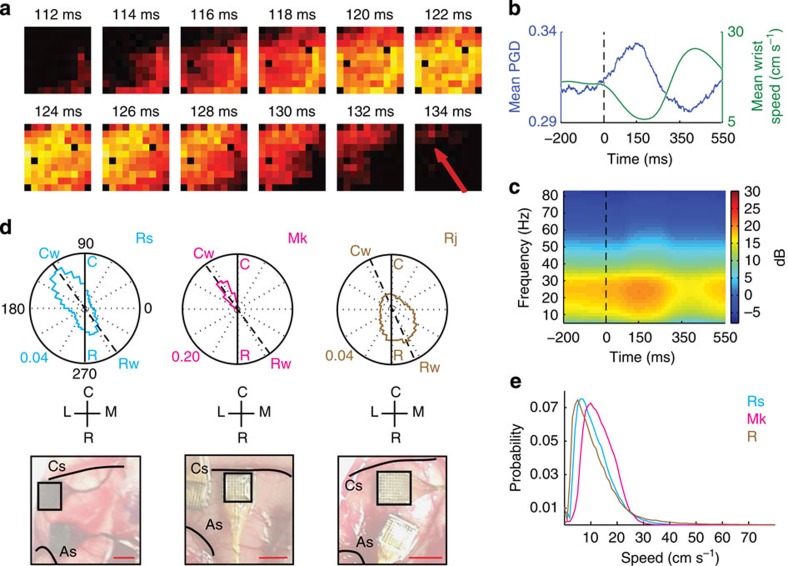
Properties of LFP beta waves. (**a**) Temporal snapshots of the LFP voltages across the array indicating wave propagation. The LFP voltage on each electrode was band-pass filtered in the beta frequency range (that is, ±3 Hz centred at the beta peak of the power spectrum). Time in milliseconds labelled above each plot is with respect to the onset of the visual target. The red arrow in the bottom right panel shows the propagation direction of the wave. (**b**) Temporal evolution of the GOF measure of planar wave activity (PGD in blue ranging from 0.295 to 0.335, as well as the mean hand speed (green) ranging from 5 to 36 cm s^−1^. (**c**) Averaged spectrogram of a single-channel LFP revealing the temporal dynamics in beta frequency power relative to the visual target onset. (**d**) Circular distributions of wave propagation directions for monkeys Rs (cyan), Mk (magenta) and Rj (brown). A solid black line denotes the rostro–caudal axis on the cortical surface. A dashed line in each rose plot connecting Cw (caudal wave direction) and Rw (rostral wave direction) denotes the axis defined by the first or only mode of beta wave propagation axis. Each panel below the circular distributions depicts the location of the multielectrode arrays (4 × 4 mm) in the arm area of MI for the corresponding subject. A red horizontal bar in the right lower corner in each panel is 4 mm. Landmarks and orientations: Cs, central sulcus; As, arcuate sulcus; C, caudal; R, rostral; M, medial; L, lateral. (**e**) Distributions of estimated propagation speeds for monkeys Rs (cyan), Mk (magenta) and Rj (brown).

We recorded multiple single-unit and LFP activity from MI using chronically implanted high-density microelectrode arrays while three rhesus monkeys (Rs, Mk and Rj) made planar reaching movements using a two-link robotic exoskeleton (BKIN Technologies, ON, Canada). The monkeys performed a random target-pursuit (RTP) task^23^ that required them to move a cursor (aligned with the position of their hand) through a sequence of randomly positioned targets. Movement durations from target to target ranged from 300 to 450 ms with mean speeds (±s.d.) of 22.33±11.17 (Rs), 14.12±6.27 (Mk) and 6.11±7.29 cm s^−1^ (Rj). Planar beta wave activity measured from spatially distributed LFP sites was evident at particular intervals of time throughout the performance of this task (Fig. 1a). We used a method described previously^13^ to characterize the properties of planar beta waves. We found that the degree of planar wave propagation as measured by a quantity called phase gradient directionality (PGD) was strongest ∼100–150 ms after the target onset (Fig. 1b) when beta power was high (Fig. 1c), and when visual target information reached the motor cortex^13^ followed by movement initiation to the new target (see wrist speed in Fig. 1b). Consistent with our previous findings using a center-out task^13^, wave propagation directions during the RTP task exhibited either a bimodal distribution (monkey Rs) or unimodal distribution with a small secondary mode (monkeys Mk and Rj), with one mode oriented in the rostral-to-caudal direction and a secondary mode oriented in the opposite direction (Fig. 1d). We denoted the caudal wave and rostral wave directions defined by the mean direction of the first or only mode of the wave propagation distribution and the opposite direction oriented roughly along the rostro–caudal axis. The distribution of propagation speeds was always unimodal with means and medians ranging from 23.2 to 26.7 and from 10.1 to 13.5 cm s^−1^, respectively (Fig. 1e).

**Figure 2:**
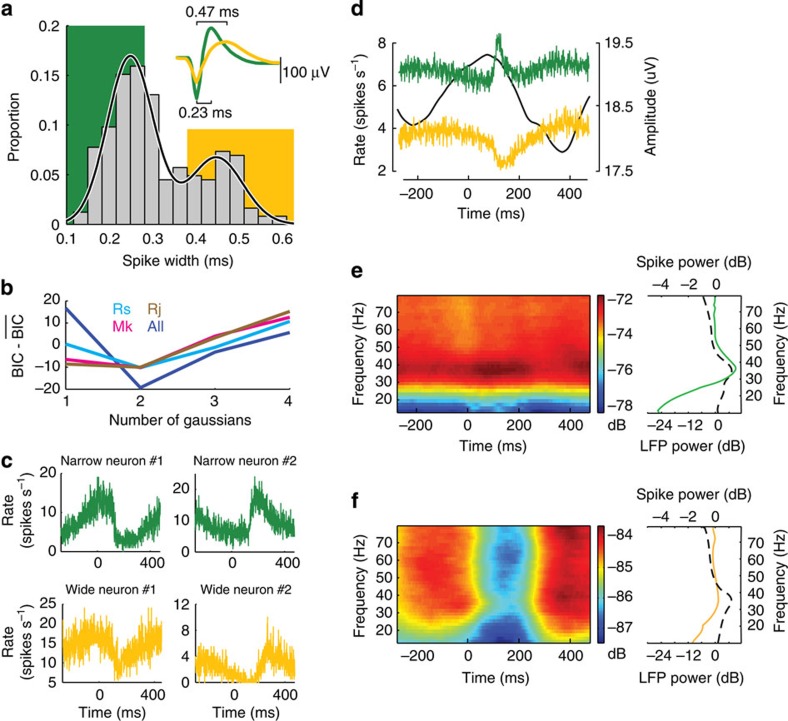
Two functional cell classes based on extracellular spike waveform width. (**a**) Histograms of spike waveform widths for all three monkeys, Rs, Mk and Rj. The solid curve indicates the fit using mixture of two Gaussians. Background colour indicates the bins classified as narrow (green) and wide (yellow) units. Inset: Examples of motor cortical units with narrow (green) and wide (yellow) spike waveform widths. (**b**) The difference between BIC values minus the mean BIC values over the range as a function of the number of Gaussians used in the mixture model to fit the distributions of spike widths. (**c**) Example peri-stimulus time histograms from two narrow (green) and two wide (yellow) spiking neurons from monkey Mk. (**d**) Averaged population spike rates for the two classes of cells from monkey Mk, narrow class in green and wide class in yellow. The time-resolved beta oscillation amplitude is plotted as a solid black line. (**e**,**f**) Averaged spectrograms for the two classes of units from monkey Mk, narrow class (**e**, left) and wide class (**f**, left), and averaged power spectra for the each class of units, narrow class in green (**e**, right) and wide class in yellow (**f**, right) computed over [−100, 300] ms relative to the visual target onset. Black dashed line on the right subfigures denotes average LFP spectrum over all channels with the base line pink noise removed.

To investigate spatiotemporal patterns of spiking activity (see Supplementary Figs. 1 and 2b,d,f for many examples of Peristimulus time histograms (PSTHs) of neurons used in the analysis), we first partitioned the population of recorded neurons into two potentially distinct, functional classes (Fig. 2a) based on the observation that their spike waveform widths exhibited a bimodal distribution. For each data set separately as well as for pooled data, a two-Gaussian mixture model (that is, bimodal) was found to be the most parsimonious fit to the distributions based on the Bayesian Information Criterion (BIC; Fig. 2b). Narrow-spiking neurons tended to exhibit higher mean spike rates as compared with broad spiking neurons for all three monkeys (however, only Mk’s data sets showed a statistically significant difference in means, unpaired two-sample *t*-test with unequal variances): 4.89±4.10 spikes s^−1^ versus 2.96±1.94 (*P*=0.0662) for Rs; 11.94±11.48 spikes s^−1^ versus 4.22±4.48 (*P*=0.0419) for Mk; and 11.68±9.00 spikes s^−1^ versus 5.59±4.45 (*P*=0.0907) for Rj. A few examples of PSTHs of neurons for monkey Mk from both narrow and wide classes used are shown in Fig. 2c (more in Supplementary Fig. 1 and for other monkeys in Supplementary Fig. 2). By summing spikes over all neurons within a class, the population spike rates of the two classes for monkey Mk were different particularly around the time that LFP beta power increased (Fig. 2d, and for other monkeys in Supplementary Fig. 2). Temporally, the spectrogram of the narrow-spiking population showed sustained power over the beta range, and the power increased when the LFP beta power increased (Fig. 2e). In contrast, the wide population exhibited a systematic power decrease in the beta and higher frequency bands around the onset of LFP beta power increase (Fig. 2f). Moreover, the spectrum of the population activity associated with narrow spike waveforms exhibited a spectral peak that was very close to the LFP beta frequency peak, whereas the population spectrum associated with wide spike waveforms exhibited no prominent beta peak (right side of Fig. 2e,f).

A number of studies have classified cortical neurons based on their extracellular spike widths, and some have suggested that narrow-spiking neurons represent local inhibitory neurons^23^,^24^,^25^,^26^,^27^,^28^. However, the range of spike widths that defined our narrow class is larger than that of many of these studies. Given that local inhibitory neurons comprise only ∼20% of all neurons in neocortex^29^,^30^, the large proportion of spiking neurons that we classified as narrow suggests that this class includes excitatory pyramidal neurons as well as local inhibitory neurons that, as a whole, have a tendency to synchronously oscillate in the beta frequency range.

### Directed effective connectivity

We developed a generative model to characterize the spiking responses of a given neuron based on the past spiking of all other simultaneously recorded neurons in the network. This model is a generalization of Granger causality to point processes, and can be used to infer excitatory- and inhibitory-directed effective connections among multiple neurons, unlike popular pairwise network estimation procedures^31^. Specifically, we estimated the conditional intensity function (CIF) of each neuron (a receiving neuron), *λ*(*t*|*H*(*t*)), given the spiking history, *H*(*t*), of all other neurons (sending neurons) in the network using a generalized linear model (GLM). The spike history from each sending neuron was composed of five 3 ms time windows for a total of 15 ms. The history length was selected based on the time it would take for the beta waves to propagate across the array at the mean estimated beta wave propagation speeds (Fig. 1e). The underlying assumptions of the model are twofold: (1) the present spike count of a receiving neuron conditioned on the past spike counts of the sending neurons is Poisson distributed, and (2) the log spike count of the receiving neuron is a linear function of the spike counts of the sending neurons. To check the sufficiency of the models used in the analysis, we performed a model-order selection analysis using the BIC, and found that the majority of the neurons showed an optimal spike history duration of <15 ms (>96%; ref. 32). To assess model goodness-of-fit (GOF) for each neuron, we used the time-rescaling theorem^33^ and Kolmogorov–Smirnov (KS) plots to define the GOF area (GOFA) and GOFA ratio (GOFR; Methods and Supplementary Fig. 3a–c). More than 85% of neurons exhibited GOFA to be <0.05 (Supplementary Fig. 3d) and >68% of neurons showed GOFR to be <0.2 (Supplementary Fig. 3e). Thus the majority of the neurons used in the analysis were reasonably well characterized as Poisson GLM.

**Figure 3:**
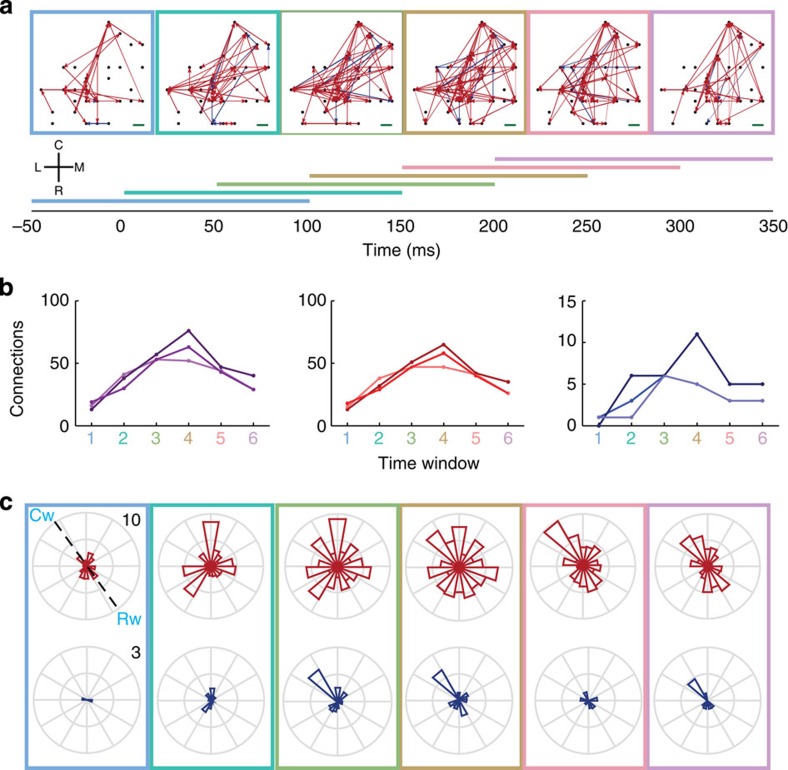
Spatiotemporal patterns of network connectivity of narrow class of neurons in MI in six 150 ms time windows incremented by 50 ms from monkey Rs data set. (**a**) Networks of significant directed connections on the array at different time windows (in milliseconds) in relation to the onset of visual target appearance. Each horizontal colour bar indicates duration of a time window used to compute a network surrounded by the same colour. C, R, M and L indicate caudal, rostral, medial and lateral orientations, respectively, on the cortical surface. Red and blue arrows represent excitatory and inhibitory connections, respectively. The black dots represent the relative positions of the electrodes on the array where the neurons were detected. Neurons whose spikes rates are ⩾1 spike s^−1^ and with narrow spike widths (≤0.267 ms) were analysed. The green scale bar on the right-bottom equals 400 μm on the cortical surface. (**b**) Number of significant connections at different time windows for both excitatory and inhibitory connections (left), excitatory connections only (center) and inhibitory connections only (right). The lines in different colour shades correspond to different time windows of data in the recording session. (**c**) Circular distribution of directed excitatory connections weighted by their strength and normalized by the total number of possible connections in each orientation on the surface of MI at the different time windows (top). Circular distribution of directed inhibitory connections weighted by their strength and normalized by the total number of possible connections in each orientation on the surface of MI at the different time windows (bottom). All rose plots are oriented in the same way as in the anatomical coordinate system defined with C, caudal; R, rostral; M, medial and L, lateral in **a**. A dashed line in the top left rose plot connecting Cw (caudal wave direction) and Rw (rostral wave direction) defines the wave propagation axis as in Fig. 1d.

**Figure 4:**
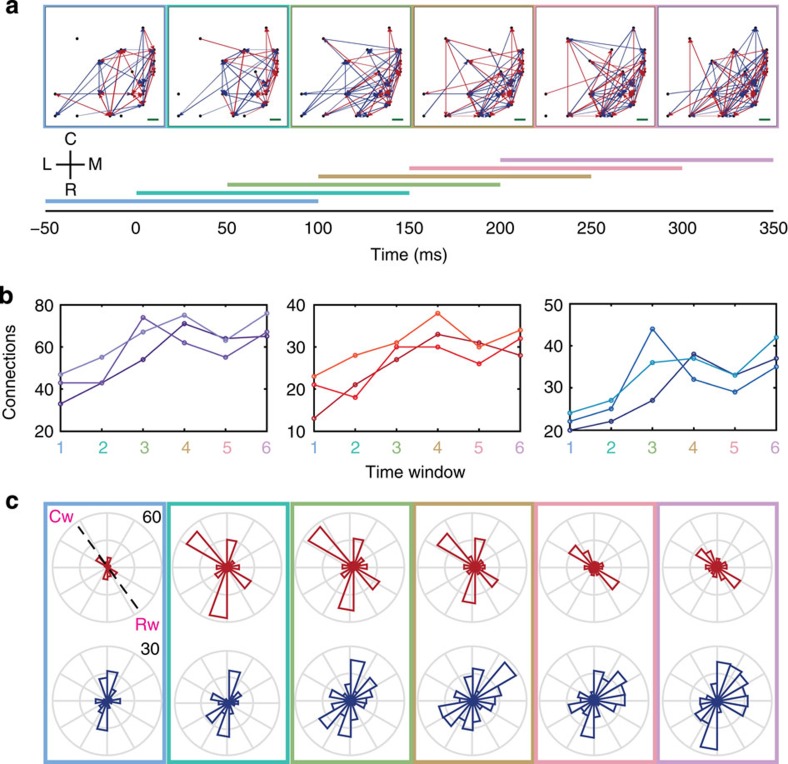
Same as Fig. 3, but for monkey Mk data set. .

**Figure 5:**
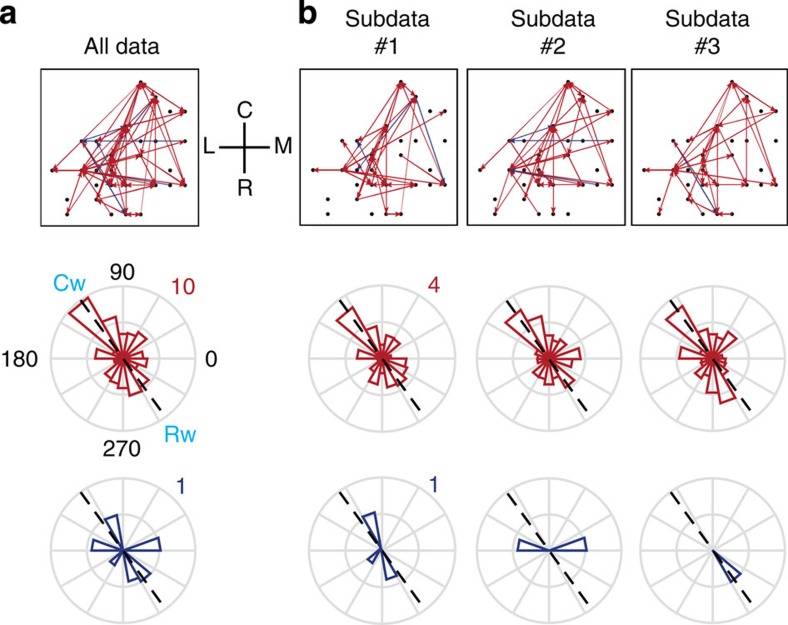
Consistency in spatial patterning of network connectivity across different subsets of data in time window 5 within the data set in monkey Rs. Networks of significant connections (top), circular distributions of excitatory connections (middle) and circular distributions of inhibitory connections (bottom). (**a**) Results from all three subsets of data were pooled. (**b**) Results from each subset of data are illustrated in each column. All plots are oriented by the coordinate system defined by C, caudal; R, rostral; M, medial and L, lateral in **a**. A dashed line in each rose plot connecting Cw (caudal wave direction) and Rw (rostral wave direction) defines the wave propagation axis as in Fig. 1d.

As in the standard Granger causality protocol, an effective connection from the sending neuron to the receiving one was inferred by comparing the log-likelihoods of the data under models with and without a particular sending neuron’s spiking history. If the performance of the model decreased significantly (*P*<0.05, *χ*^2^-test) when a sending neuron was removed, an effective connection was inferred, and the sign of the sum of the five history terms determined whether the connection was excitatory or inhibitory^34^. We estimated the complete network of significant directed connections between recorded neurons with narrow spike waveforms and mean spike rates of at least 1 Hz (to ensure numerical stability) in different time windows. The temporal evolution of the networks in 150 ms windows incremented by 50 ms steps is illustrated in Fig. 3a for monkey Rs with 38 narrow neurons, Fig. 4a for monkey Mk with 21 narrow neurons and Supplementary Fig. 4a for monkey Rj with 33 narrow neurons. The largest number of connections was observed between 100 and 350 ms after the appearance of a new visual target at a time slightly after LFP beta power and the PGD reached their peak values. The temporal evolution of network topology estimates, based on the number of excitatory, inhibitory and all connections, was highly reliable as determined by calculating networks on non-overlapping subsets of data within each data set (Fig. 5 and Supplementary Fig. 5). Furthermore, in order to check whether our connectivity results depended on the precise spike timing among neurons or were a consequence of rate modulation (neurons with very high spike rates are more likely to have effective connections), we randomly shuffled the trial order for each neuron and within each of eight target directions, and recomputed network interactions. We found that the number of connections were significantly lower as compared with the unshuffled networks (Supplementary Fig. 6 for monkeys Rs and Mk), suggesting that effective connections reflect single-trial coordination across neurons, rather than overall rate modulations or tuning properties.

To place the estimated networks in a spatial context, we computed the orientation of each connection on the cortical surface. For each cortical orientation, we summed up each effective connection weighted by its strength (as determined by the log-likelihood ratio, see Methods section), and normalized the sum of the weighted connections by the total number of possible connections for that orientation. This normalization was performed to account for anisotropies in the spatial arrangement of the recorded neurons. Between −50 and 100 ms relative to visual target onset, few connections were observed and very little spatial structure was evident. The number of connections increased in subsequent epochs, and peaked between 50 and 250 ms after the onset of a new visual target. The clear bimodality in the circular distribution of the excitatory connection network emerged between 150 and 250 ms after the visual target onset (Fig. 3c for monkey Rs, Fig. 4c for monkey Mk and Supplementary Fig. 4c for monkey Rj, and Supplementary Fig. 7). Furthermore, this bimodality remained even when we used one spike-width threshold value, the weighted average of the means of the two Gaussians fit on all spike widths (Supplementary Fig. 8).

**Figure 6:**
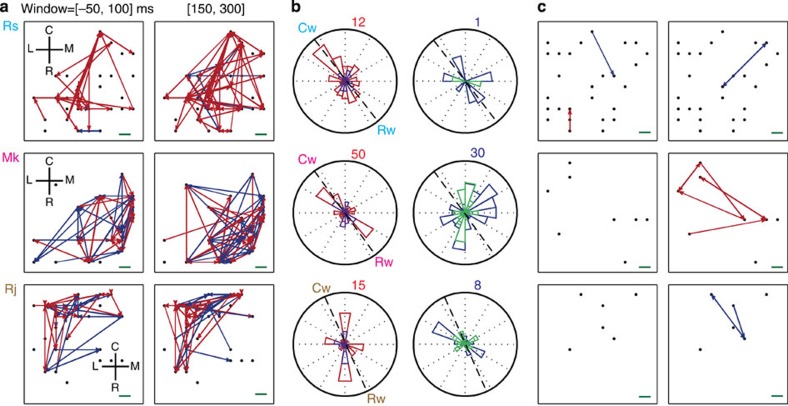
Spatiotemporal patterns of network connectivity of narrow and wide classes of neurons in two time windows. Data from monkeys Rs (top), Mk (middle) and Rj (bottom). (**a**) Networks of significant directed connections on the array at different time windows. Red and blue arrows represent excitatory and inhibitory connections, respectively. The black dots represent the relative positions of the electrodes on the array where the neurons were detected. If >1 neuron was detected on a given electrode, a given dot contains all units on that electrode. Neurons with firing rates ⩾1 spike s^−1^ and narrow spike widths (≤∼0.2667, ms) in MI were analysed. The green scale bar on the right-bottom equals 400 μm on the cortical surface. R, rostral; C, caudal; M, medial and L, lateral denote the anatomical orientation. (**b**) Circular distributions of directed excitatory (left) and inhibitory (right) connections weighted by their strength and normalized by the total number of possible connections. The circular distribution of excitatory connections for time window [−50, 100] ms in purple overlies that for time window [150, 300] ms in red. The circular distributions of inhibitory connections for the same windows are in teal and blue, respectively. The dashed black lines represent the beta wave axis. (**c**) Same as in **a**, but using neurons with firing rates ⩾1 spike s^−1^ and wide spike widths (⩾0.4000, ms).

By comparing the network topology during early and late time windows (Fig. 6a, left and right respectively), we observed the emergence of a larger number of significant connections and a strong anisotropy of directed excitatory connections. The circular distribution of excitatory connection directions became bimodal and closely oriented to the beta wave axis during the later time window in all three monkeys. By fitting the beta wave directional distributions with a mixture of two von Mises distributions, we determined the mode and the circular s.d. (CSD). We found that the modes of excitatory connections fell within 1.1 times the CSD of the beta wave modes. In particular, the beta wave primary/secondary modes measured with respect to the anatomical medial direction (±CSD) versus excitatory connection modes were 123/279±34/52 degrees versus 130/290 degrees (Rs), beta wave modes of 120/258±18/60 degrees versus 140/50 degrees (Mk) and beta wave modes of 297/104±72/51 degrees versus 270/90 degrees (Rj; Fig. 6b, left). The circular distributions of inhibitory connection directions were also bimodal but oriented almost orthogonal to the beta wave axis (Fig. 6b, right). In contrast to the narrow-spiking neurons, there were few, if any, statistically significant connections among wide-spiking neurons in each time window (Fig. 6c).

For excitatory connections between narrow-spiking neurons, we took the mean of the five time delays weighted by their model coefficient values, and computed the average time delay between each excitatory connection. Dividing the distance of each excitatory cell pair projected along the beta wave axis by the average time delay provided an estimate of the ‘conduction’ velocity of each excitatory connection along the beta wave axis. Estimated velocities ranged from 10 to 30 cm s^−1^ across all six time windows and four data sets, in rough agreement with our estimated wave propagation speeds.

### Spatial anisotropy and target information

**Figure 7:**
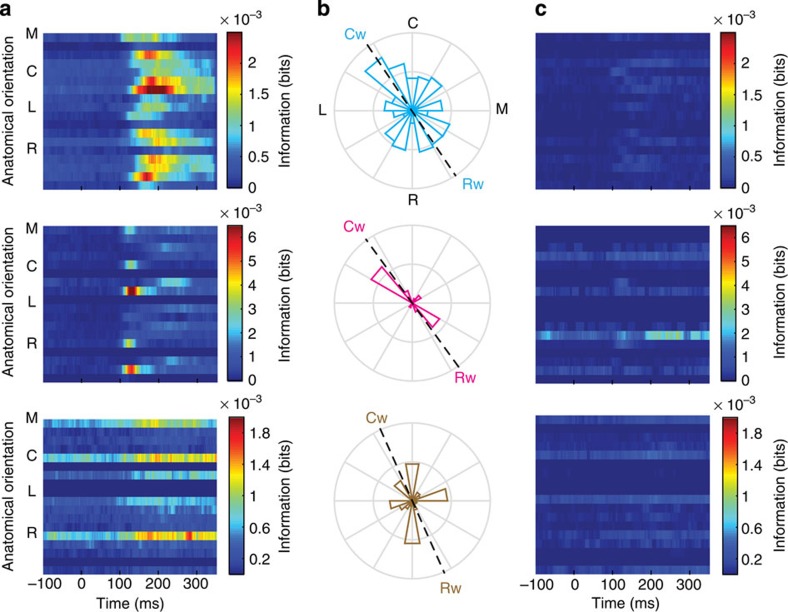
Target-direction information content of spike sequencing between neurons with narrow spike waveforms. Data from monkeys Rs (top), Mk (middle) and Rj (bottom). (**a**) Time-resolved, mutual information between spike sequencing and target direction (that is, the direction from the location of the acquired target to the new target). Information was calculated for only pairs of neurons with excitatory connections. The colour map consists of 18 rows corresponding to 18 orientations (20-degree bins) aligned to anatomical orientations denoted with M, medial; C, caudal; L, lateral and R, rostral, with information in bits in blue-red false colour. (**b**) Circular distributions of spike sequencing information with respect to the orientation of neuron pairs on the cortical surface. Spike sequencing information was summed over the time window in [100, 350] ms and over pairs within each orientation and then normalized. The dashed black lines represent the beta wave axis defined by Cw (caudal wave direction) and Rw (rostral wave direction) as in Fig. 1d. (**c**) Target-direction information content of sequencing firing of non-connected narrow-spiking neurons.

In order to investigate any behavioural relevance to the spatial anisotropy of excitatory connections among narrow neurons, we next investigated the information content of the sequential spiking activities between neuron pairs with excitatory connections. Sequential spiking activity at time *t* was defined as the product of the receiving neuron’s activity at time *t* and the integrated activity of the sending neuron’s activity in the 15-ms time interval preceding time *t*. We found systematic differences in sequential spiking activity as a function of target direction. To assess this quantitatively, we calculated the mutual information between target direction and the sequential spiking of narrow neuron pairs that possessed excitatory effective connections as a function of time (Fig. 7a). For each of 18 cortical orientation bins, the information was summed across all cell pairs with excitatory connections and divided by the total number of possible connections in that orientation. It should be noted that we added information across cell pairs in order to capture relative information trends in time and across different cortical orientations. Absolute information values cannot be inferred because addition assumes a lack of redundancy across cell pairs, an assumption that may not be warranted. Spike sequencing provided peak target-direction information ∼150–250 ms for monkey Rs, 100–150 ms for monkey Mk and 150–300 ms for monkey Rj. The circular distribution of mutual information exhibited spatial anisotropy whose peaks were oriented along the beta wave axis, 130 degree for monkey Rs, 140 degree for monkey Mk and 90 degree for monkey Rj with respect to the medial direction, and they were all within the beta wave primary mode direction±CSD (Fig. 7b). Cross-correlation coefficient peaks (within ±1 orientation bin) between the mutual information and beta wave distributions were 0.520, 0.353 and 0.252 for monkey Rs, Mk and Rj, respectively. Spike sequencing of narrow-spiking neurons that were not effectively connected showed an order of magnitude of smaller amount of total mutual information compared with those pairs with excitatory effective connections, and the spatial pattern of information did not resemble the distribution of beta wave directions (Fig. 7c). To verify that this spatial pattern of information was specific to the sequential firing of neuron pairs, we computed target-direction information from single-unit spiking activity (Supplementary Fig. 9a,b). The circular distribution of orientations of neuronal pairs weighted by the mean single-neuron mutual information values averaged over the two neurons with an excitatory connection between them revealed a weaker alignment to the beta wave axis (Supplementary Fig. 9c) than that of mutual information of sequential spiking of neurons with excitatory connections. Cross-correlation peaks (within ±1 orientation bin) between mean single-neuron information and beta wave distributions were −0.165, −0.135 and 0.102 for monkey Rs, Mk and Rj, respectively (Supplementary Fig. 9d,e).

## Discussion

Propagating wave activity is ubiquitous throughout the cortex but has never been characterized at the single-neuron level. Although our results do not directly demonstrate that single units propagate their spiking activity in a wave-like manner across the motor cortical sheet, we show for the first time spatiotemporal spike patterning that closely matches propagating wave activity as measured by LFPs in terms of both its spatial anisotropy and its transmission velocity. Although the horizontal anisotropy of directed effective connections among the narrow-spiking neurons has never before been experimentally documented (however, effective anisotropy was used in a modelling study^22^), anatomical data on the horizontal connectivity of motor cortex may shed light on these observations. By staining degenerating axons and terminals after localized lesions within MI, Gatter and Powell documented a preponderance of horizontal axons in layers 2/3 and 5 in non-human primates that were more spatially extensive along the rostro–caudal dimension as compared with the medio–lateral dimension^35^. In addition, Huntley and Jones^36^ used retrograde labelling injections in primate motor cortex and found weak strips of labelled cell bodies arranged in the rostro–caudal dimension. However, these data do not provide evidence for stronger (anatomical or functional) connections in the rostro–caudal dimension.

Physiological data also suggest that the functional connections we observed may reflect properties of horizontal connectivity within motor cortex. Electrical stimulation and current-source density analysis in rat motor cortical slices found long-distance excitatory, monosynaptic interactions mediated by horizontal connections in layers 2/3 and 5 with a conduction velocity of ∼10 cm s^−1^, which is on the same order of magnitude as the propagation velocities we documented with the LFP wave and the excitatory connections in our effective connectivity analysis^37^. Based on spatially smoothed multiunit responses recorded from the auditory cortex, propagating activity was documented with comparable propagation speeds of ∼20 cm s^−1^ using a similar gradient field method^11^.

Although the beta wave propagation axes and propagation speeds were consistent across all the animals used in the study, there are two issues to be resolved in future studies. First, the orientation of the primary beta wave direction was not consistent across the three monkeys. It is not obvious why this would be the case, but it may be owing to the exact placement of the arrays in the medial/lateral dimension along the motor strip. Second, our monkeys exhibited a fairly wide range of hand movement speeds while wave propagation speeds were fairly consistent. We would like to investigate in more detail whether there is a relationship between the kinematics, kinetics and muscle activity of the limb, and the statistical properties of the beta waves.

Our observation that population activity among narrow-spiking neurons exhibited a pronounced beta spectral peak suggests that beta oscillations in the motor cortex may be mediated by a network of functionally distinct cells^38^. Simulation studies have suggested that beta oscillations emerge in network interactions of local inhibitory neurons^22^, which in turn entrain excitatory pyramidal neurons to fire rhythmically^39^. These local inhibitory and excitatory pyramidal cells may represent the population of narrow-spiking neurons that tend to oscillate at the beta frequency range. In contrast, wide-spiking neurons as a population do not resonate at any particular frequency, and, moreover, exhibit a reduction in oscillatory power in the beta and higher frequency bands around the time at which LFP beta power increases.

The temporal dynamics of planar wave activity, spatially oriented effective connections and motor behaviour indicate the following chain of events. The degree of planar wave activity begins increasing at target appearance and reaches a maximum at 100–150 ms later. Thus, this enhancement of planar wave activities in the beta oscillation range appears to be triggered by arrival of information about the visual target in MI. At roughly 150 ms after target onset, the rostro–caudal anisotropy of excitatory effective connections emerges (Figs 3 and 4, and Supplementary Fig. 4), and sequential firing among these connected neurons begins carrying information about target direction. Finally, movement speed begins to increase 200 ms after target appearance resulting in movement to the target. It is our view that spatiotemporal dynamics of the LFP provides a mesoscopic perspective of patterning that is generated by spatiotemporal arrangements among groups of single units. Therefore, planar wave activity reflects the underlying spatiotemporal distribution of spiking activity among the units. A recent modelling study^22^ showed spatial anisotropy among inhibitory cells, while our study showed spatial anisotropy in excitatory connections. However, a recent study using an immature cerebral cortex preparation showed that cortical waves in layer III were blocked by glutamatergic receptor antagonists, but not by GABAergic receptor antagonists^40^. Furthermore, a modelling study showed that long-range coherent oscillations with some delay were partially induced by interactions of excitatory cells^41^.

These results may have implications for the development of cortically controlled brain machine interfaces. Current decoding algorithms for brain machine interfaces have ignored the spatial organization of cortical neurons and their functional interactions. In principle, by augmenting the GLM framework, we used here by including external movement covariates, we could invert these spike prediction models to decode movement parameters. Furthermore, a recent study has speculated that spatiotemporally patterned electrical stimulation in motor cortex (similar to the recorded patterns we have observed in our study) could replace deep brain stimulation to treat symptoms of Parkinson’s disease^42^.

Our information analysis of sequential firing may have important behavioural implications. First, we have shown that functionally connected, narrow-spiking neurons carry more task-relevant information in their sequential firing than do unconnected, narrow-spiking neurons. This implies that there is a subnetwork of neurons within the motor cortex that may be playing a special role in representing and transmitting information to guide behaviour. Second, this behavioural information is most pronounced in the sequential firing of neurons that are spatially aligned along the rostro–caudal axis, implying that the motor cortex possesses information-carrying, spatial structure beyond somatotopy. Future work will investigate whether downstream targets such as the muscles are sensitive to and may take advantage of this spatial structure when driving motor behaviour.

## Methods

### Behavioural tasks

Three male macaque monkeys (*Macaca mulatta*) were operantly trained to move a cursor appearing above the monkey’s hand location to targets projected onto a horizontal, reflective surface in front of the monkey. The monkey’s arm rested on arm troughs secured to links of a two-joint exoskeletal robotic arm (BKIN Technologies, ON, Canada) underneath the projection surface. The shoulder joint was abducted 90 degrees such that shoulder and elbow flexion/extension movements were made in the horizontal plane. The shoulder and elbow joint angles during movements were sampled at 500 Hz by the robotic arm’s motor encoders. The position of the hand was computed using the forward kinematics equations. The monkeys performed the random RTP task, in which a sequence of seven targets appeared on the projection surface. At any one time, a single target appeared at a random location in the workspace, and the monkey was required to move the cursor to it. As soon as the cursor reached the target, the target disappeared and a new target appeared in a new, random location. After reaching the seventh target, the monkey received water reward.

### Electrophysiology

A silicon-based electrode array composed of 100 electrodes (1.0 mm electrode length and 400 μm interelectrode distance) was implanted in the arm area of MI of each monkey. During a recording session, signals from up to 96 electrodes were amplified (gain, 5,000), band-pass filtered between 0.3 and 7.5 kHz, and recorded digitally (14-bit) at 30 kHz per channel using a Cerebus acquisition system (Blackrock Microsystems, Inc., UT). Only waveforms (1.6 ms in duration; 48 sample time points per waveform) that crossed a threshold were stored and spike sorted using Offline Sorter (Plexon, Inc., TX). Signal-to-noise ratios were defined as the difference in mean peak-to-trough voltage divided by twice the mean s.d. The mean s.d. was computed by measuring the s.d. of the spike waveform at each of the 48 sample time points of the waveform and then averaging. All isolated single units used in this study possessed signal-to-noise ratios of 3:1 or higher. The data for each neuron were converted to a binary time series at a 1-ms time resolution.

A total of four data sets (one data set for animal Rs, two data sets from animal Mk, which were 4 days apart, and one data set for animal Rj,) were analysed, where a data set is defined as all simultaneously recorded neural data collected in one recording session. Each data set contained between 59 and 115 simultaneously recorded units from MI. A total of 302 single-unit samples were recorded from MI over all four data sets. We use the term ‘samples’ because the recordings were made from chronically implanted arrays in each monkey. Therefore, data collected over recording sessions in monkey Mk are not necessarily from completely different ensembles of units. Ensembles consisted of ‘randomly’ selected units from MI except for a possible bias for neurons with large cell bodies that would generate higher signal-to-noise ratios. All of the surgical and behavioural procedures were approved by the University of Chicago’s Institutional Animal Care and Use Committee and conform to the principles outlined in the Guide for the Care and Use of Laboratory Animals (NIH publication no. 86-23, revised 1985).

### Beta wave characterization

We used the wave characterization method as defined in Rubino *et al*.^13^. Briefly, we first applied a band-pass filter bidiretionally whose center frequency for each data set was defined to be the most prominent peak frequency over the beta oscillation range (10–40 Hz) and the width was ±3 Hz, then applied the Hilbert transform to a filtered voltage signal recorded by an electrode located at (*x*,*y*) on the array *V*(*x*,*y*,*t*) to obtain the instantaneous amplitude *A*(*x*,*y*,*t*) and phase as *φ*(*x*,*y*,*t*) follows:




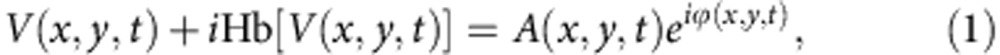




where Hb is the Hilbert transform operator and *A*(*x*,*y*,*t*) is the instantaneous amplitude of the voltage signal, and *i*^2^=−1. The wave velocity ***v***(*t*)=[d*x*/d*t*,d*y*/d*t*] was computed by taking the derivative of a constant contour of




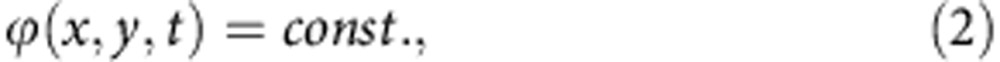




with respect to time:




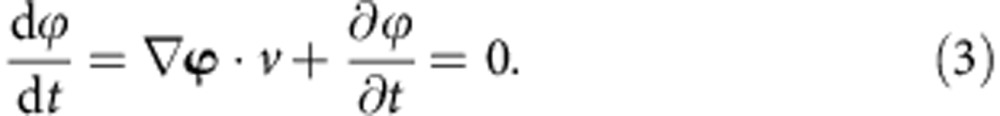




Since the direction of the velocity, −∇*φ*, is perpendicular to the constant phase contour, the speed is




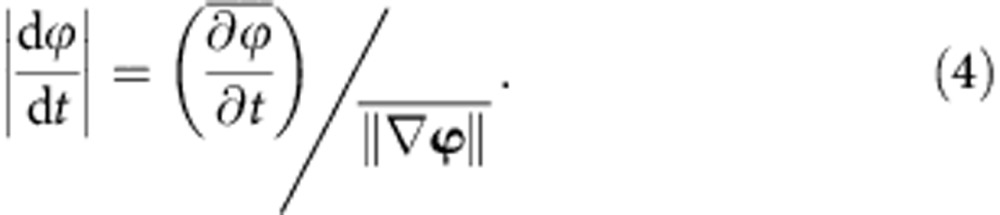




Note that velocity is well defined only when the phase gradient is non-zero and when the signal exhibits a well-defined propagation direction. In order to measure how well the phase gradients align across the array, we used the quantity called the PGD(*t*):




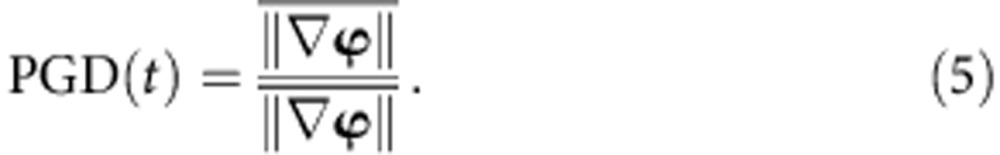




The bar here denotes the spatial average. If the phase gradients at all spatial locations align at a given time, *t*, then PGD(*t*)=1. If the phase gradients are randomly distributed, then PGD will be close to 0. Thus, we used a threshold value of 0.2, which is based on the tail of the PGD distribution when channel assignments are randomly shuffled^43^. As long as PGD(t) was above the threshold, then we used the estimated values of mean wave direction and speed across the array, which were computed as:




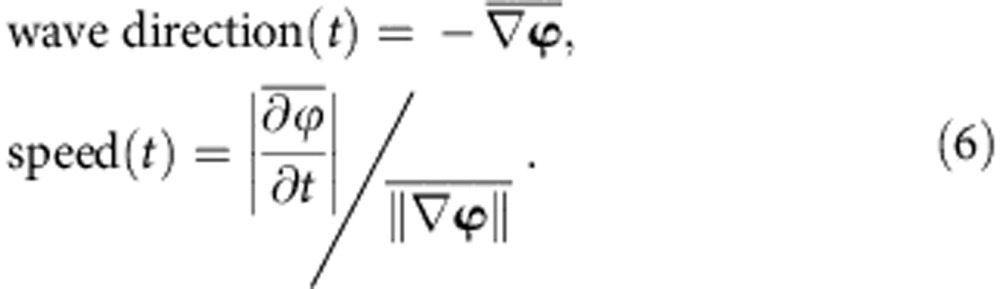




### Unit spiking data selection

We created trial-based data sets using a time interval −100 to 350 ms relative to visual target onset. We had 2,547, 2,746, 1,999 and 1,526 trials from each of the four data sets. We first selected only neurons whose average firing rates during the interval of interest, −100 to 350 ms relative to visual target onset, were higher than 1 spike s^−1^. Then, we fitted a mixture of Gaussians to the distribution of waveform widths where the width was defined as the duration from trough-to-peak of the mean waveform of each sorted unit. We used mixture Gaussian models from 1 to 4 Gaussians for each data set and the pooled data. Then, we computed the BIC for each data set and the pooled data set for each model to find a most parsimonious Gaussian mixture model. Every data set and the pooled data set had a minimum BIC at 2 (Fig. 2b). Thus, we decided to fit each data set with a mixture of two Gaussians. For each data set, we computed the average of the two means from the two Gaussians. The smallest average of all data sets fell between 0.2667 and 0.3000, ms, and neurons with waveform widths of 0.2667, ms or less were classified as narrow-spiking units. Similarly, the largest average of the two means fell between 0.3667 and 0.4000, ms so that neurons with waveform widths of 0.4000, ms or more were classified as wide-spiking units. Thus, we had two thresholds. We decided to take this conservative threshold scheme to clearly separate the two populations of neurons. The numbers of neurons that satisfied the narrow condition were 38 neurons (4.89±4.10 spikes s^−1^, mean±s.d.) among a total of 115 neurons for the first data set (monkey Rs), 14/21 neurons (11.94±11.48 and 9.71±8.59 spikes s^−1^, respectively) among a total 59/65 neurons for the second and third data sets (monkey Mk), and 33 neurons (11.68±9.00 spikes s^−1^) among a total of 63 neurons for the fourth data set (monkey Rj). The numbers of wide-spiking neurons were 29 neurons (2.96±1.94 spikes s^−1^) among a total of 115 neurons for the first data set (monkey Rs), 13/10 neurons (4.22±4.48 and 4.88± ±5.69 spikes s^−1^, respectively) among a total of 59/65 neurons for the second and third data sets (monkey Mk), and 6 neurons (5.59±4.45 spikes s^−1^) among a total of 63 neurons for the fourth data set (monkey Rj). Neurons that did not fall into either the narrow or wide class were not further analysed. Peri-stimulus time histograms binned for two example neurons used in the analyses are presented based on the spike-width class for each data set in Fig. 2c, and Supplementary Figs 1 and 2. We also broke up each data set into three subsets of data consisting of an equal number of trials for the first two monkeys, and into two subsets of data for the third monkey to check for temporal consistency. We analysed each trial using 150 ms time window.

### Power spectra and spectrograms for spiking of population of neurons

We binned each neuron’s spike train into 1 ms bins and summed the binned spike trains over all neurons within the narrow or wide spike waveform population. Then, we used a multitaper spectrum estimation method^44^ (time-bandwidth product and number of tapers were 3 and 5, and moving window of 250 ms in 2 ms increments) to compute average power spectra over [−100, 300] ms relative to the visual cue onset and average spectrograms over the time windows specified above using Chronux toolbox.

### Granger causality measure for point process models

We utilized a model-based methodology for analysing the causal interactions of multiple neural spike trains^34^. The discrete, all-or-nothing nature of a sequence of action potentials together with their stochastic structure suggests that neural spike trains may be regarded as point processes^45^,^46^. Let *N*_*i*_(*t*) denote the sample path that counts the number of spikes of neuron *i* in the time interval (0,*t*] for *t* ∈ (0,*T*] for *i*=1,…,*M* recorded neurons. A point process model of a spike train for neuron *i* can be completely characterized by its CIF, *λ*_*i*_(*t*|*H*(*t*)), defined as:




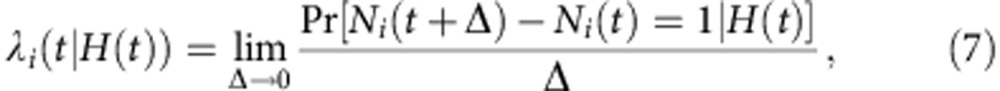




where *H*(*t*) denotes the history of the spiking activity of all neurons up to time *t*. The CIF represents the instantaneous firing probability and serves as a fundamental component for constructing the likelihoods and probability distributions for point process data. It is a history-dependent function, and reduces to a Poisson process if it is independent of the history. To simplify the notation, we denote *λ*_*i*_(*t*|*H*(*t*)) as *λ*_*i*_(*t*). We used a point process GLM for building models whose history terms contain past spiking activity of other neurons^34^. To test whether neuron *j* causes neuron *i* in the ‘Granger’ sense^47^, we developed two classes of point process GLMs for neuron *i* : one given by *P*(*N*_*i*_(0:*T*)) that has the past of all neurons as the covariates for the CIF, and the other given by *Q*(*N*_*i*_(0:*T*)) that has the past of all except for neuron *j*. The point process likelihood is given by^45^




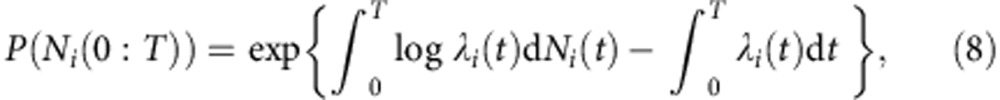




where




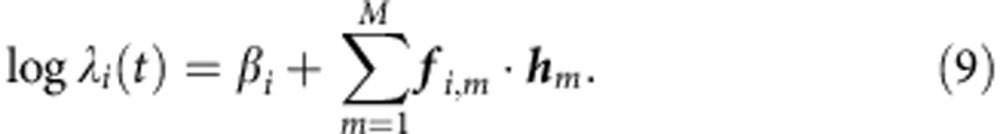




In equation (9), we used a point process GLM to model the relationship between the spiking activity and its covariates (the spiking history)^45^. Here, *β*_*i*_ relates to a background level of the activity of neuron *i*, ***f***_*i*_,_*m*_ is a 5-dimensional (5D) vector of parameters that relate the past spiking of neuron *m* to the current spiking of neuron *i*, and ***h***_*m*_ is a 5D vector each of whose elements counts the spikes in every 3 ms time window of 15 ms spiking history of neuron *m*, these parameters were chosen to obtain a relatively small number of parameters while maintaining the necessary temporal resolution. The ‘·’ represents the dot product between vectors. Note that for *P*(*N*_*i*_(0:*T*)), *λ*_*i*_(*t*) includes the past spiking of all neurons. The other point process likelihood, *Q*(*N*_*i*_(0:*T*)), is given by the same equation (8), but with *λ*_*i*_(*t*) replaced with 
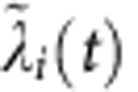
:




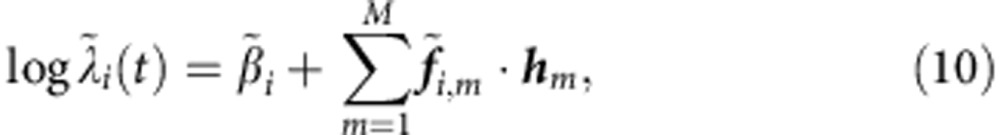




which excludes the effect of the past spiking of neuron *j*. Model parameters *θ*_*i*_={*β*_*i*_,***f***_1,1_,…,***f***_1,*M*_} for *P*(*N*_*i*_(0:*T*);*θ*_*i*_) and 
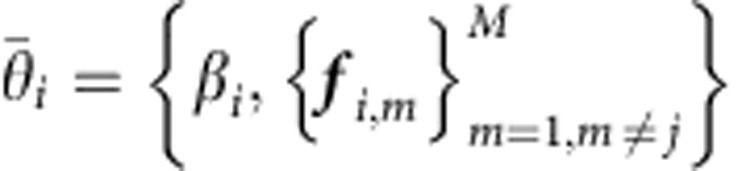
 for 
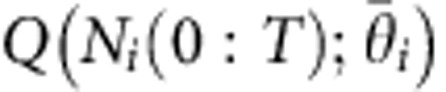
were fitted by maximizing the likelihoods^48^, and they were calculated by




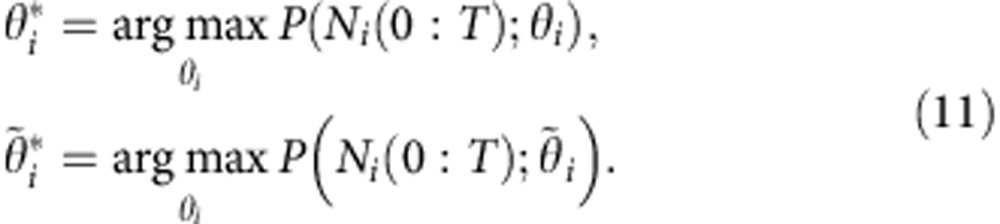




Because of the structure of the equation (8), the maximum likelihood solution can be efficiently calculated. Finally, we define our causality measure from *j* to *i* as




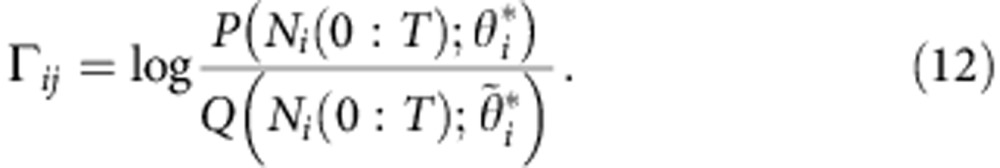




If the spiking history of neuron *j* helps predict the spiking activity of neuron *i*, the log-likelihood ratio should be significantly greater than zero, and then we say that neuron *j*, ‘Granger-causes’ *i*^49^. The equality of the likelihood ratio holds when neuron *j* has no causal influence on *i*. Excitatory and inhibitory influences from neuron *j* to *i* are distinguished by the sign of 
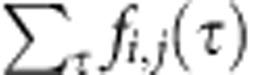
 in equation (9), which represents the summed influence of the past spiking of *j* on *i*.

### Significance test

The Granger causality measure, Δ*D*=*D*_0_−*D*_1_=2Γ_*ij*_ given by the log-likelihood ratio provides an indication of the relative strength of causal interaction but gives little insight into whether or not it is statistically significant. We use a statistic based on the log-likelihood ratio test to address this issue. We denote the deviance obtained using the model parameter 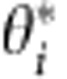 as *D*_0_, and the deviance obtained using 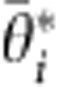 as *D*_1_. The deviance difference between two models is equivalent to two times log-likelihood ratio given by Δ*D*=*D*_0_−*D*_1_=2Γ_*ij*_ (ref. 50). If both models describe the data well, then the deviance difference may be asymptotically described by 
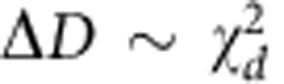
 where *d* is the difference in dimensionality of two models, and *d*=5 in our study. If the value of Δ*D* is in the critical region, that is, greater than the upper tail 100(1−*α*)% of the 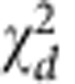 where *α* determines false-positive rates, then the causal influence is determined as statistically significant. We used a multiple-hypothesis testing error measure called false discovery rate to address the multiple comparison problem^51^.

### Assessment of GOF against the data for the point process models

The validation of a designed statistical model for data is required before making an inference based on the model. The time-rescaling theorem was used to transform point process neural spike train data to a continuous measure appropriate for GOF assessment^33^. Once a CIF is estimated, rescaled times can be computed using the estimated CIF. These rescaled times will be uniformly distributed random variables on the interval (0,1] if the estimated CIF is a good approximation to the true conditional intensity of the point process. To evaluate whether the rescaled times followed the uniform distribution, we ordered these rescaled times from the smallest to the largest, and then plotted the quantiles of the cumulative distribution function of the uniform distribution on (0,1] against the ordered rescaled times. This form of graphical representation is termed a KS plot. If the model is consistent with the data, then the points should lie on a 45-degree line. Approximate 95% confidence bounds for the degree of agreement between the model and the data may be constructed using the distribution of the KS statistic. We defined the GOFA bounded by the 45-dergee line and the cumulative distribution in the KS plot. We also defined the GOFR computed as the ratio of GOFA to an area out of the ∼95% confidence bounds for the degree of agreement (see Supplementary Fig. 3a for schematics of the method). These measures were computed for each neuron in each 150 ms time window.

### Causal inference with shuffled trials

We estimated causality networks after shuffling the trial order of each neuron for the same target direction using the data after the onset of visual cue. Through this analysis, we tested how much the precise spiking times among multiple neurons contributed to significant functional interactions between them. If significant functional interactions were simply caused by an increase in firing rate after the onset of visual cue, shuffling the trials would not affect the network estimates. On the other hand, if they were caused rather by the increase of relative spike timings among multiple neurons, shuffling the trials would break the relative spike timings among neurons and thus lead to a sparser network estimate.

For this analysis, we used the data set in the window 5 (after the onset of cue) of monkeys Mk and Rs. For each target direction, we permuted the order of all the trials for each neuron and then estimated the causality network using the shuffled data set. We repeated this procedure 100 times. The mean number of connections was 2.05 with an s.d. of 1.79 compared with 56, 65 and 66 connections for monkey Mk, and the mean number of connections was 12.40 with an s.d. of 1.50 compared with 100, 105 and 128 connections for monkey Rs from the original unshuffled network. This analysis suggests that the increase of significant interactions between neurons after the onset of visual cues is caused by more organized spike sequencing among neurons rather than an increase of spiking rate.

### Mutual information analysis for single-unit spiking activities and spike sequencing

We evaluated the information content of single-unit spiking activities and spike sequencing using the mutual information between the target direction and single-cell spiking, or spike sequencing between two neurons for each cortical orientation, respectively. Mutual information between two variables represents the reduction in uncertainty of one variable due to the knowledge of the other. It is defined as




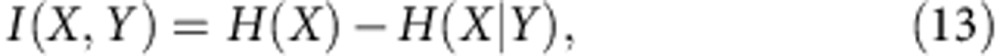




for random variables *X* and *Y* where *H*(*X*) is the Shannon entropy of *X* (ref. 52). In this paper, *Y* is a random variable representing the target directions, and takes on one of eight directions (the eight octants in 2D plane) during the RTP task. For single-neuron analysis, *X* represents the binary sequence of unit spiking activity. For spike sequence analysis, *X* represents the spike sequencing from neuron *i* to *j* defined by




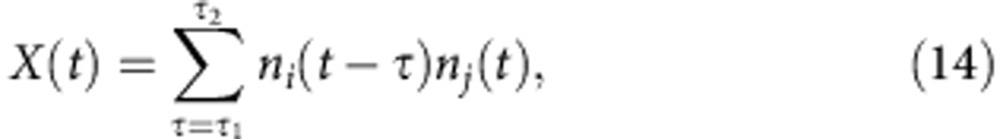




which is calculated as the sum of products in spike counts between neuron *i* at varying time lags *τ* (from *τ*_1_ to *τ*_2_) and neuron *j*. For the spike sequencing, we used *τ*_1_=1 ms and *τ*_2_=15 ms. We chose a 15-ms summation window to match with the duration of history terms used for the GLM described above. Information content of the spike sequencing was calculated using the empirical distributions of *X*(*t*) at fixed *i* and *j*, and *t* over all trials for different target directions. Using the position information of neurons on the multielectrode array, we calculated the cortical orientation of the information content of spike sequencing. To account for biases in estimating information from limited data, the target direction labels were randomly shuffled over all trials so that a trial originally associated with target direction *k* could be randomly assigned one of the eight target directions. Mutual information between target direction and spike sequencing was then calculated. The average information from 10 shuffles was subtracted from the estimated information values.

All the numerical analyses were performed using Matlab (Mathworks, MA). Matlab codes for Granger analysis are available at the website (http://www.neurostat.mit.edu/gcpp).

## Additional information

**How to cite this article:** Takahashi, K. *et al*. Large-scale spatiotemporal spike patterning consistent with wave propagation in motor cortex. *Nat. Commun.* 6:7169 doi: 10.1038/ncomms8169 (2015).

## Supplementary Material

Supplementary Information Supplementary Figures 1-9
