# Supplementary material for: Large-scale spatiotemporal spike patterning consistent with wave propagation in motor cortex
Source: Nat Commun. 2015 May 21;6:7169. doi: 10.1038/ncomms8169 (PMC4443713; doi:10.1038/ncomms8169)
Supplement: Supplementary Information — Supplementary Figures 1-9 [file ncomms8169-s1.pdf]

## Supplementary Materials

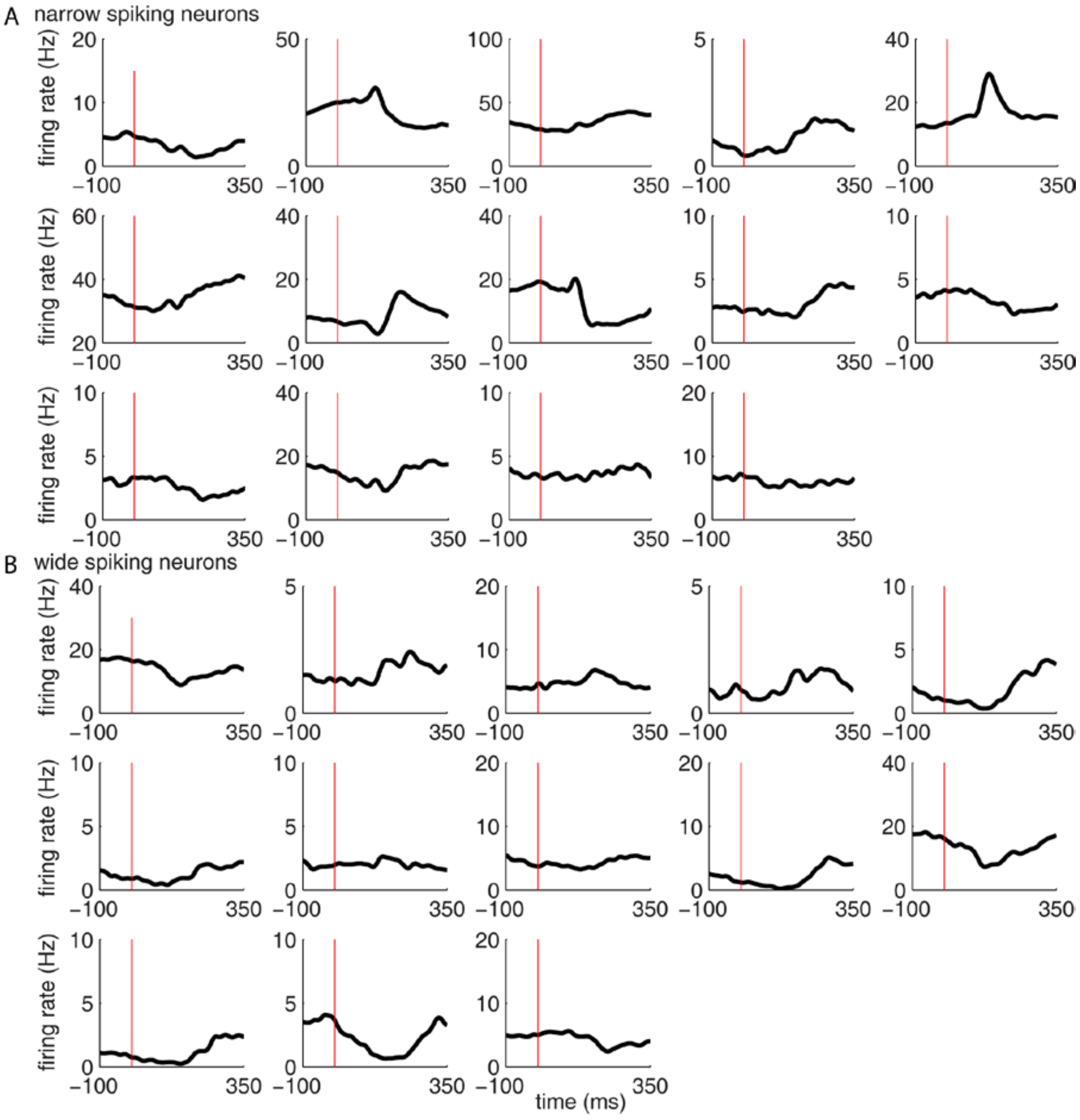


**Supplementary Figure 1. Peri-target time histograms (PSTHs) of neurons from narrow and wide classes for monkey Mk.** Top half shows examples of PSTHs for narrow neurons and bottom half shows examples for wide neurons.

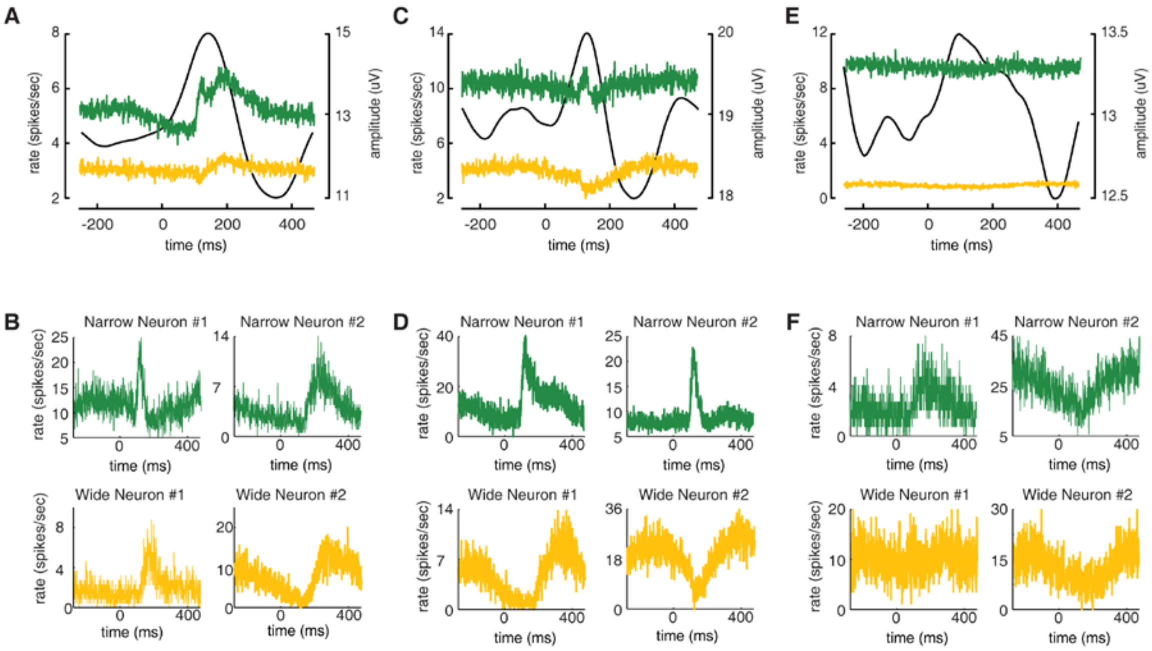


**Supplementary Figure 2. Two functional cell classes based on extracellular spike waveform width.** Averaged population spike rates for the narrow class (green) and wide class (yellow) of cells from monkey Rs (A), from monkey Mk's second data set (C), and from monkey Rj (E). The time-resolved beta oscillation amplitude is plotted as a solid black line. Example peri-stimulus time histograms of two narrow and two wide spiking neurons from monkey Rs (B), from monkey Mk's second data set (D), and from monkey Rj (F).

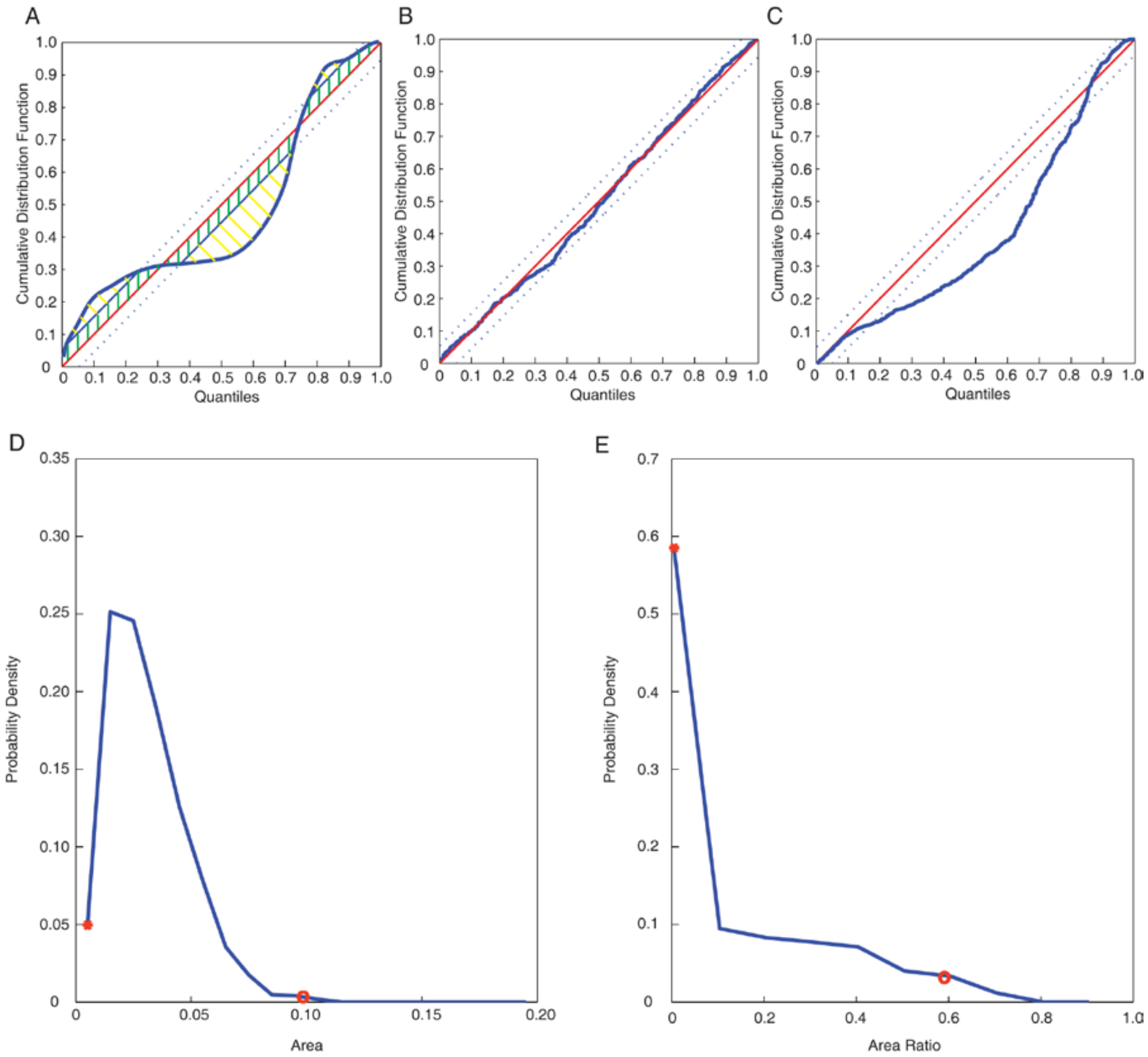


**Supplementary Figure 3. Goodness-of-fit of generalized linear models of individual neurons based on KS plots obtained using the time rescaling theorem.** (A) Schematics of definitions of goodness-of-fit areas (GOFA) and goodness-of-fit area ratios (GOFR). GOFA is defined to be the area between the 45-degree line (red) and the KS plot (blue). GOFA is divided into two areas by the approximate 95 % confidence intervals (blue dashed). Thus, GOFA is the sum of the areas shaded by green lines and yellow lines. GOFR is defined to be the ratio of the area shaded by yellow lines to GOFA. (B) An example of a KS plot of a neuron that is well characterized by the model, GOFA=0.0102, GOFR=0 (C) Another example of a KS plot of a neuron that is not well characterized by the model. GOFA=0.0984, GOFR=0.5969. (D) Distribution of goodness-of-fit areas (GOFA) for all neurons over the all four data sets. Red star and circle denote the neurons in (B) and (C) respectively. (E) Distribution of goodness-of-fit area ratios (GOFR) for all neurons over all four data sets. Red star and circle denote the neurons in (B) and (C) respectively.

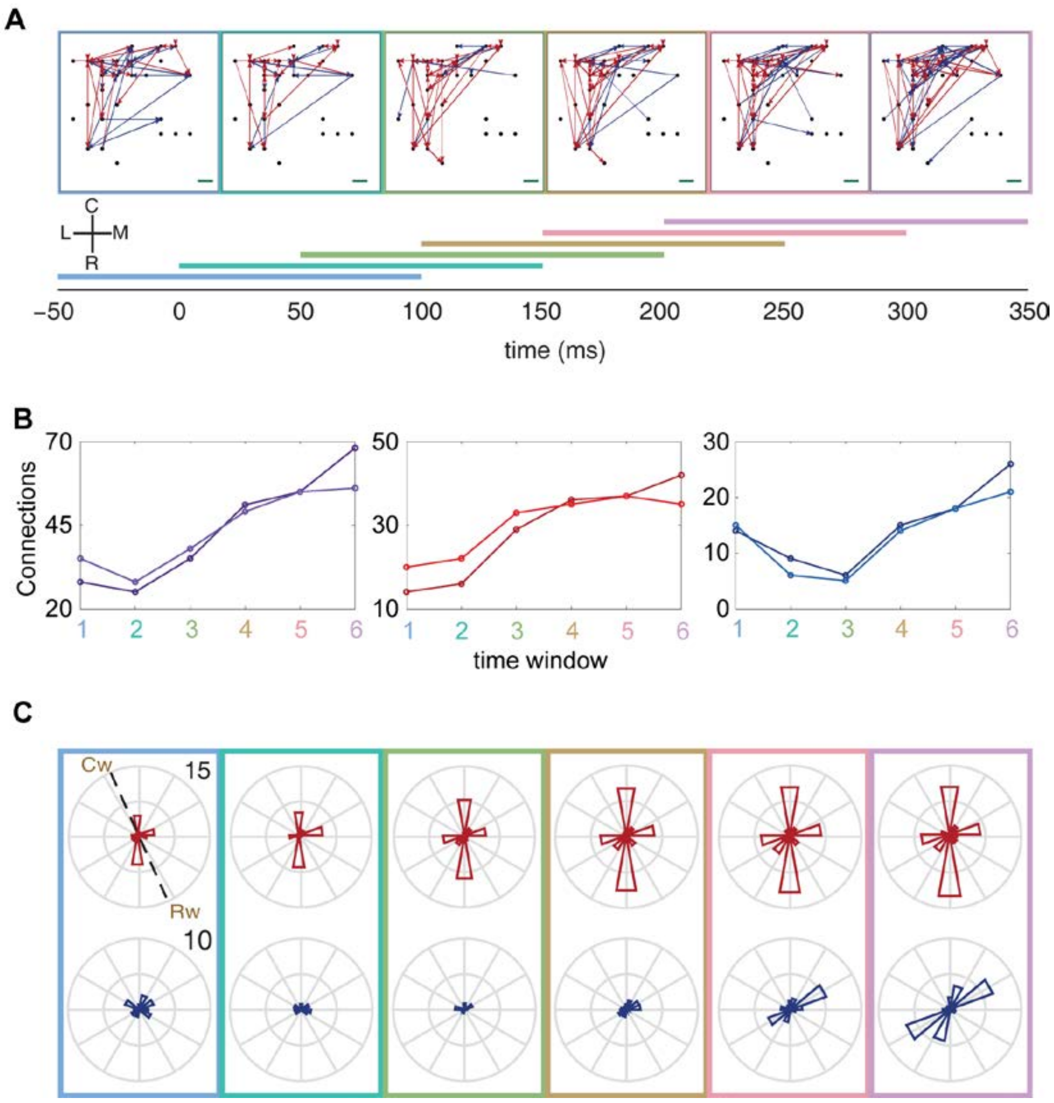


**Supplementary Figure 4. Spatiotemporal patterns of network connectivity in MI of monkeys Rj.** Same as in Fig. 3 and 4 for monkeys Rs and Mk, but using a dataset from monkey Rj.

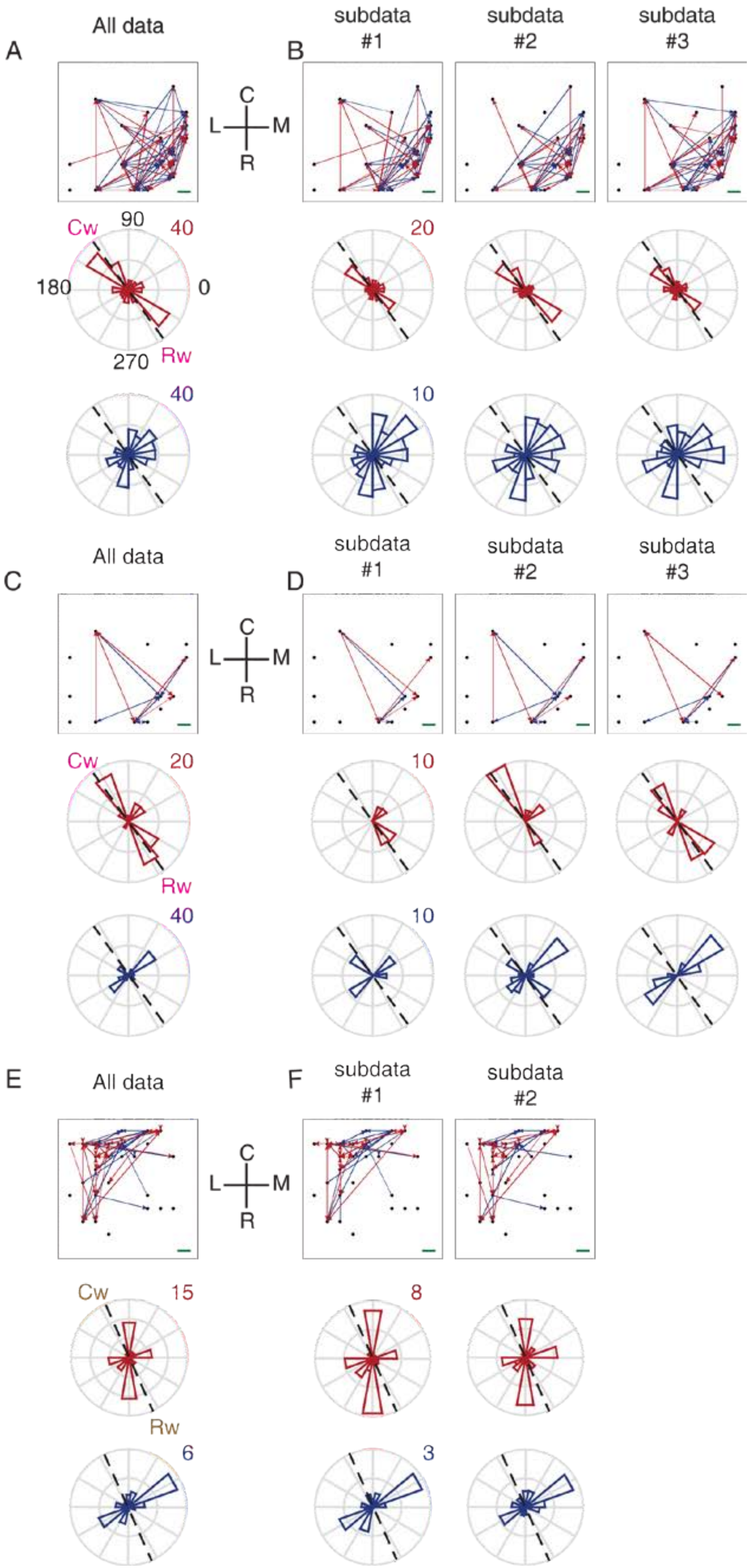
A
All data
B
subdata #1
subdata #2
subdata #3
C
L
M
R
Cw
90
40
180
0
270
Rw
20
40
10
C
All data
D
subdata #1
subdata #2
subdata #3
Cw
20
Rw
10
40
10
E
All data
F
subdata #1
subdata #2
Cw
15
Rw
8
6
3

**Supplementary Figure 5. Consistency in spatial patterns of network connectivity across different subsets.** Same as Fig. 5. For each monkey, networks of significant connections (top), circular distribution of excitatory connections (middle), and circular distribution of inhibitory connections (bottom). (A) and (B) for one data set from monkey Mk, (C) and (D) for another data set from monkey Mk, and (E) and (F) for monkey Rj. In each rose plot**,** a dashed line connecting Cw (Caudal wave direction) and Rw (Rostral wave direction) is the same as in Fig. 1 D for each animal.

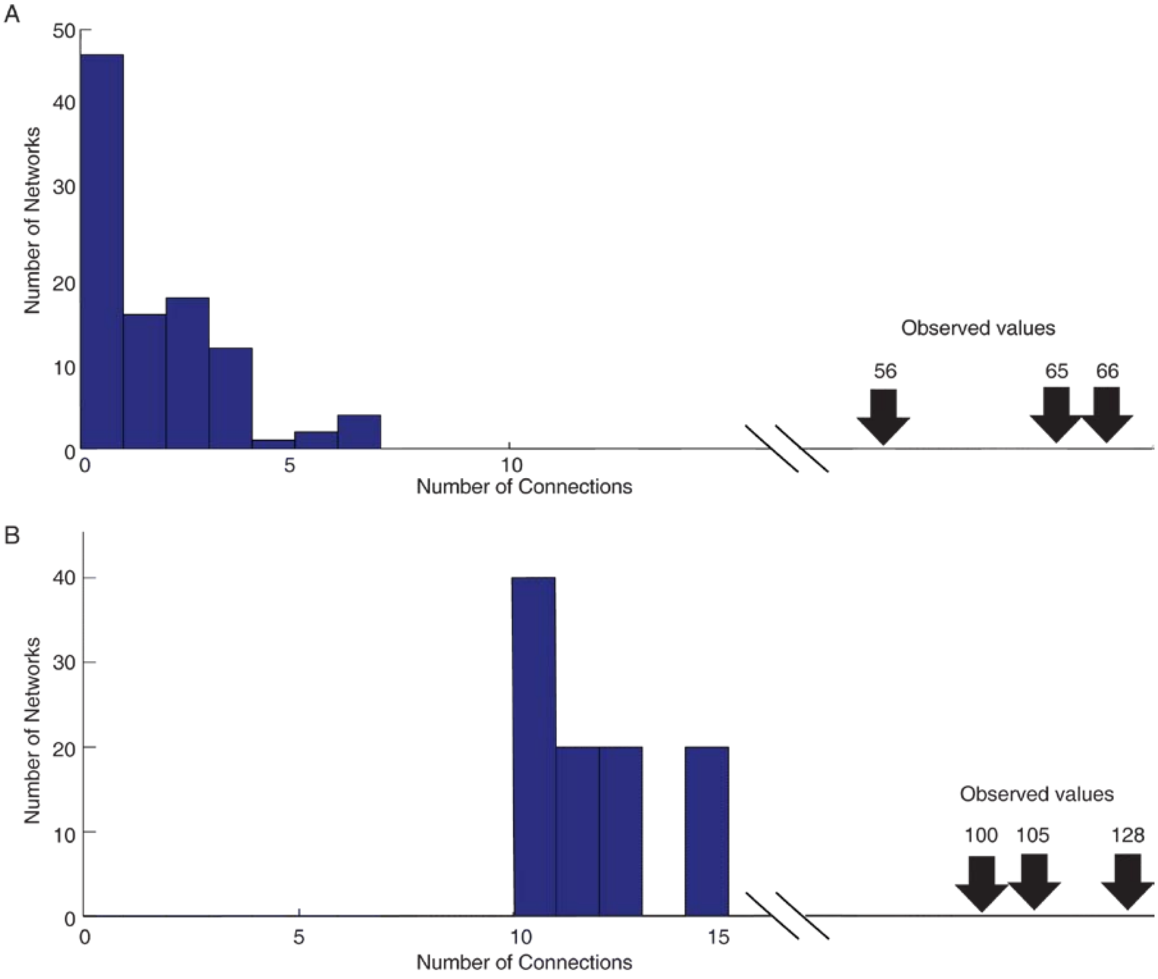


**Supplementary Figure 6. Shuffle analysis.** Using time window 5 from Mk and Rs datasets, the trial order was randomly shuffled 100 times for each neuron within each of 8 target directions to compute 100 corresponding networks. (A) The numbers of connections for all shuffled networks for monkey Mk were significantly lower (mean = 2.05 with a standard deviation of 1.79) than those of three unshuffled subsets of data (65, 56, 66). Furthermore, most of the shuffle networks did not show consistent directional anisotropy as was observed with the unshuffled data. (B) Same as (A) for monkey Rs. The numbers of connections for all shuffled networks were significantly lower (mean = 12.40 with a standard deviation of 1.50) than those of three unshuffled subsets of data (100, 105, 128).

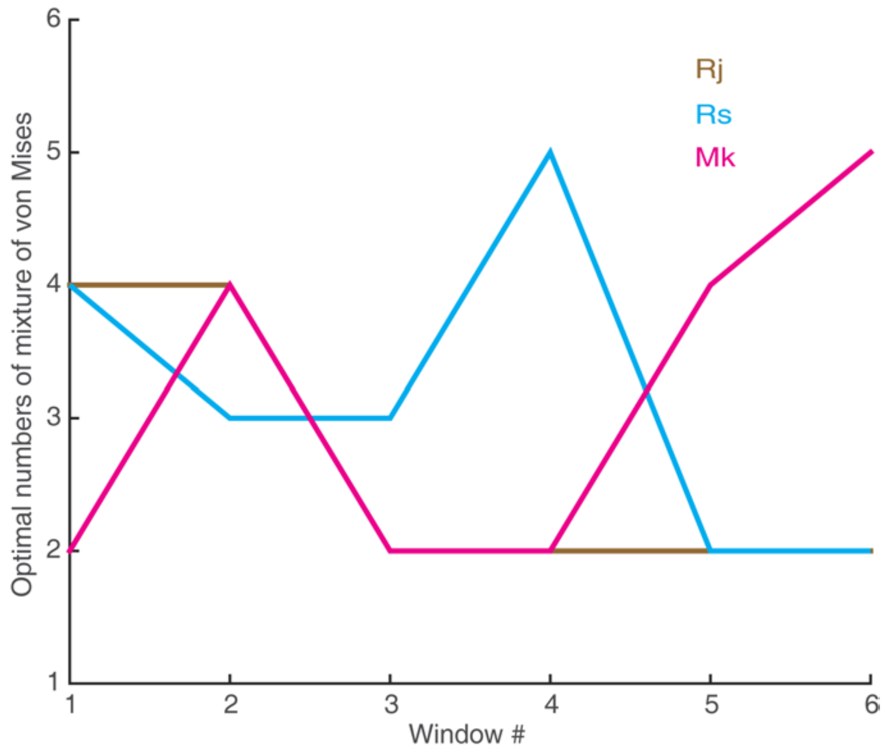


**Supplementary Figure 7. The optimal number of von Mises distributions for weighted connection direction distributions of excitatory connection among narrow neurons.** The optimal number of von Mises distributions is computed based on Akaike Information Criteria (AIC) for each time window to characterize weighted circular distributions of the spatial orientations of directed excitatory connections of narrow neurons for each monkey, Rs (cyan), Mk (magenta), and Rj (brown).

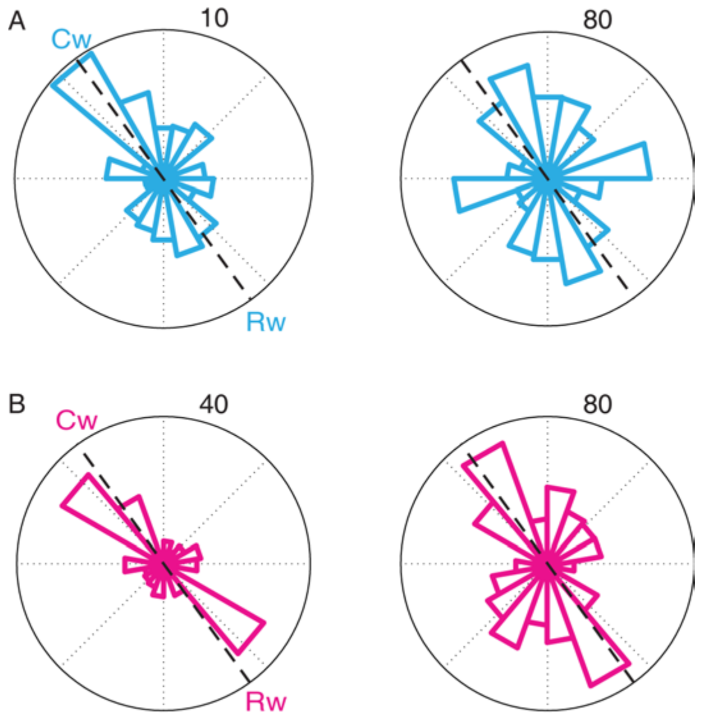


**Supplementary Figure 8. Difference in topology of narrow-spiking, excitatory networks using a conservative and more liberal spike-width threshold.** (A) Monkey Rs - Left: Same as the top left panel of Fig. 6B with 38 narrow spiking neurons using a spike-width threshold of 0.267 ms. Right: Distribution of weighted excitatory connections with 48 narrow spiking neurons using a spike-width threshold of 0.300 ms. (B) Monkey Mk - Left: Same as the middle left panel of Fig. 6B with 21 narrow spiking neurons with a spike-width threshold of 0.267 ms. Right: Distribution of weighted excitatory connections with 25 narrow spiking neurons using a spike-width threshold of 0.300 ms. A dashed line connecting, in each rose plot, Cw (Caudal wave direction) and Rw (Rostral wave direction) is the same as in Fig. 1 D for each animal.

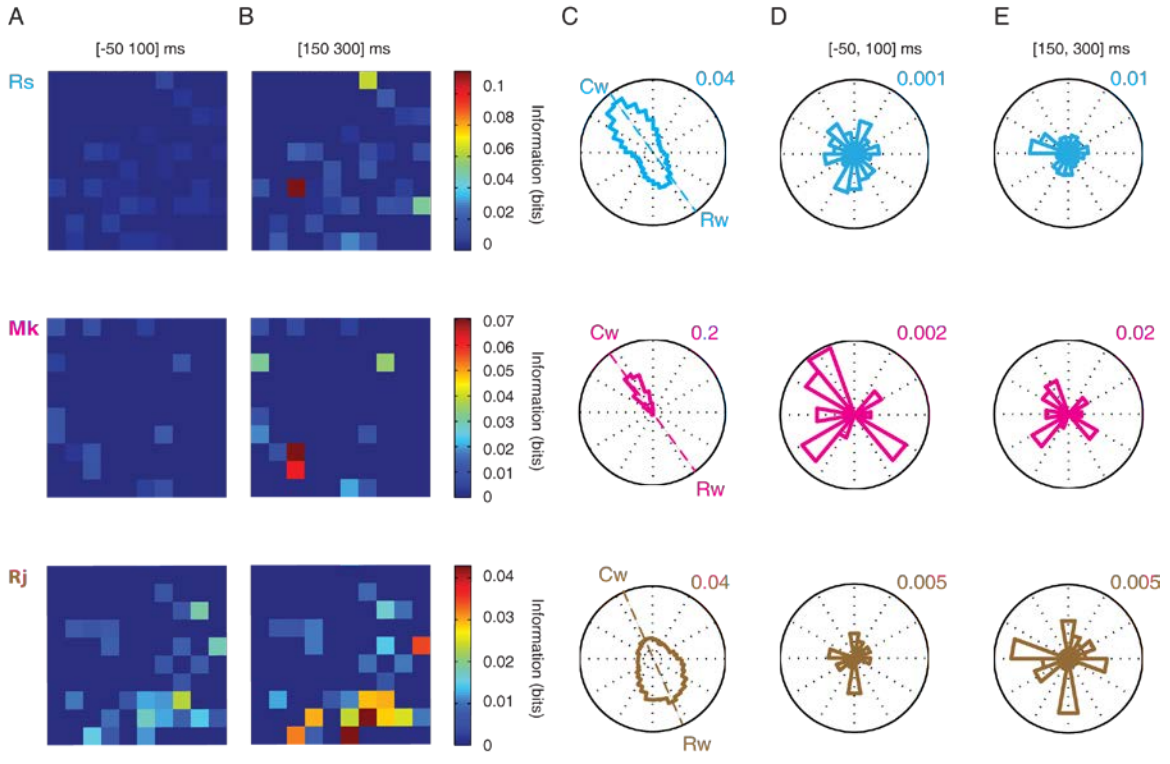


**Supplementary Figure 9. Spatiotemporal patterns of target-direction information content in firing rates of single units and pairs of units with narrow spike waveforms.** (A,B) Information content in firing rates of single units computed over the two time windows [-50,100] (A) and [150, 300] (B) relative to the visual target onset for one data set from each monkey used in the current study Rs (top), Mk (middle), Rj (bottom). Each square corresponds to the array location of the electrode on which t he unit was recorded and shows the mutual information between the unit's firing rate and target direction in color. (C) LFP beta wave propagation distributions for the corresponding data sets from Fig 1(C) are shown with Cw and Rw as defined in Fig. 1(D). (D,E) Orientation distributions of the average information of pairs of narrow spiking neurons. The single neuron information was averaged over each pair of cells and then summed over all pairs for a given orientation and then normalized by the total number of pairs for that orientation. Only units that exhibited excitatory connections based on our network analysis are included. Distributions are computed over the two time windows [-50,100] (D) and [150, 300] (E) relative to the target onset for one data set from each monkey in the current study Rs (top), Mk (middle), Rj (bottom).
